# It Is Advisable to Control the Duration of Hypothermia Circulatory Arrest During Aortic Dissection Surgery: Single-Center Experience

**DOI:** 10.3389/fcvm.2021.773268

**Published:** 2021-12-10

**Authors:** Jian Song, Jinlin Wu, Xiaogang Sun, Xiangyang Qian, Bo Wei, Wei Wang, De Wang, Jiawei Qiu, Fangfang Cao, Wei Gao, Rui Zhao, Lu Dai, Shuya Fan, Enzehua Xie, Juntao Qiu, Xinjin Luo, Cuntao Yu

**Affiliations:** ^1^Department of Vascular Surgery, National Center for Cardiovascular Diseases, Fuwai Hospital, Chinese Academy of Medical Sciences and Peking Union Medical College, Beijing, China; ^2^Department of Cardiac Surgery, Guangdong Provincial People's Hospital, Guangdong Cardiovascular Institute, Guangdong Academy of Medical Sciences, Guangzhou, China; ^3^Department of Cardiac Surgery, Fuwai Hospital, National Center for Cardiovascular Diseases, Chinese Academy of Medical Sciences and Peking Union Medical College, Beijing, China; ^4^Department of Intensive Care Unit, Fuwai Hospital, National Center for Cardiovascular Diseases, Chinese Academy of Medical Sciences and Peking Union Medical College, Beijing, China

**Keywords:** aortic dissection, arch surgery, hypothermia circulatory arrest, prognosis, aortic surgery

## Abstract

**Objective:** The duration of hypothermic circulatory arrest (HCA) is one of the important factors affecting the prognosis of arch surgery, which is still controversial. The purpose of this study was to investigate the effect of HCA duration on early prognosis in type A aortic dissection (TAAD) patients who underwent arch surgery in our center.

**Methods:** All consecutive patients who underwent surgical treatment for TAAD in Fuwai Hospital from January 2013 to December 2018 were included in this study and divided into four quartile groups based on HCA time. Baseline characteristics, perioperative indicators, and early mortality were statistically analyzed by propensity score matching (PSM) and restricted cubic spline (RCS) method. Perioperative adverse events were confirmed according to the American STS database and Penn classification.

**Results:** About 1,018 consecutive patients (mean age 49.11 ± 1.4 years, male 74.7%) with TAAD treated surgically were eventually included in this study. After PSM, with the prolongation of HCA time, the surgical mortality rates of group [2,15], (15,18], (18,22], and (22,73] were 4.1, 6.6, 7.8, and 10.9% with *p* = 0.041, respectively. As shown in RCS, the mortality rate increased sharply after the HCA time exceeded 22 min. And from the subgroup analysis, the HCA time of 22 min or less was associated with better clinical outcomes (OR 2.09, 95%CI 1.25–3.45, *p* = 0.004).

**Conclusions:** The early mortality increases significantly with the duration of HCA time when arch surgery was performed. And multiple systems throughout the body can be adversely affected.

## Introduction

Acute aortic dissection (AAD) is a life-threatening disease characterized by high morbidity and mortality and remains a challenge in its diagnosis and treatment, especially in type A aortic dissection (TAAD). Although the in-hospital or 30-day mortality rate has decreased in recent years (from 31 to 22%) with the improvement of surgical techniques and strategies, the long-term overall mortality rate remains high (57%) (1, 2). Total arch replacement (TAR) is the most difficult and challenging among all kinds of surgical procedures for TAAD.

Hypothermia circulatory arrest (HCA) provides the surgeon with a safe time to perform arch reconstruction surgery. Combined with unilateral or bilateral cerebral perfusion, the clinical effect and safety of total arch surgery can be significantly improved (3, 4). HCA can lead to organ ischemia, neurological disorders, and long-term high mortality (5). In order to reduce the damage caused by HCA, hybrid aortic surgery and aortic balloon occlusion (ABO) were introduced, but the clinical results of these techniques did not bring better benefits compared with traditional total arch surgery (6–9).

Many aortic surgery centers have focused on the effects of different core temperatures and assisted cerebral perfusion strategies on surgical outcomes (10–14), while few studies have addressed the effect of duration of HCA on the overall patient population. Our team has also recently studied HCA temperature and brain protection strategies demonstrating that moderate HCA (MHCA) combined with antegrade cerebral perfusion (ACP) is a safe and effective technique for TAAD (15). Therefore, the purpose of this study was to clarify the relationship between HCA time and early postoperative outcomes based on our single-center clinical experience.

## Methods

### Patients and Definition

All consecutive patients who underwent surgical treatment for TAAD in Fuwai Hospital (Beijing, China) from January 2013 to December 2018 were included in this study. Patient inclusion criteria are as follows: (1) TAR was performed for DeBakey I aortic dissection and (2) adopt HCA technology. A total of 1,018 TAAD patients were included in the final analysis. The study was approved by the Chinese Ethics Committee, with informed consent not required due to its observational nature (Reference Number: ChiECRCT-20180041).

According to the experience and operation habits of our center, the core temperature maintained at 20–24°C is defined as deep HCA (DHCA), and the temperature maintained at 24-28°C is defined as MHCA. About 1,018 patients were divided into four quartile groups based on HCA time. HCA time between 2 min (inclusive) and 15 min (inclusive) was defined as [2,15] group, between 15 min and 18 min (inclusive) was defined as (15,18] group, and so on remaining two groups, i.e., (18,22], (22,73] groups, were defined.

The definition of complications involved in this study was based on the definition of the American STS database. The classification of organ malperfusion is based on patients' condition at the time of presentation according to the Penn classification (16). Surgical mortality is defined as a composite of in-hospital and 30-day mortality.

### Operative Techniques

The surgical methods of TAR in our center have been described in detail in previous articles (17). The flow rate of cerebral perfusion was maintained to 8 ml/kg/min. Systemic perfusion was resumed after completion of the distal aortic arch anastomosis or using ABO to block blood flow, at which point HCA ended. Finally, anastomosis of the left common carotid artery, the proximal ascending aorta, the innominate artery, and the left subclavian artery was completed successively.

### Statistical Analysis

Statistical analysis and data visualization were performed using R 3.6.1 (R Foundation for Statistical Computing, Vienna, Austria). For continuous variables, a normal distribution test was first carried out. The normally distributed data were expressed as mean ± SD, and a *t*-test was used for comparison between groups. The abnormal distribution data were expressed as median and range interquartile, while Fisher's test was used for comparison between groups. Categorical variables were presented as frequencies with percentages and analyzed by the chi-square test or Fisher's exact test, as appropriate. A two-tailed *p* < 0.05 was considered statistically significant.

To adjust for unbalanced basic characteristics, a propensity score matching (PSM) analysis was performed. The preoperative variables in the four groups were performed with PSM, and the *P*-value of the preoperative variable after matching was close to 1. Then, the preoperative and postoperative variables were compared between the groups. A restricted cubic spline (RCS) was used to flexibly model and visualize the relation of HCA time with surgical mortality. Further, a subgroup analysis was performed by temperature, gender, age, year of operation, acuity, and Penn classification, respectively.

## Results

About 1,018 consecutive patients (mean age, 49.1 ± 11.4 years; male, 74.7%) with TAAD treated surgically were admitted to the Fuwai Hospital. Patients in the acute stage before surgery accounted for 84%. Preoperative Marfan's syndrome (MFS) and other family histories of aortic disease were identified in 91 (8.9%) and 20 (2.0%) patients, respectively. According to Penn classification, 761 (74.8%) was Penn Aa, 229 (22.5%) was Penn Ab, 21 (2.1%) was Penn Ac, and 7 (0.7%) was Penn Abc. The overall baseline data are shown in Supplemental Table 1. The distribution of surgical strategies in the overall cohort is as follows: 22 (2.2%) patients underwent TAR, 916 (90.0%) patients underwent TAR with frozen elephant trunk (FET), and 80 (7.9%) patients were treated with the ABO technique. The mean CPB time was 171.8 ± 47.6 min, the mean HCA time was 18.3 ± 7.2 min, and the mean nasopharyngeal temperature was 22.8 ± 3.5°C. Overall operation data are shown in Supplemental Table 2. There were 72 (7.1%) patients who died before discharge or within 30 days (Supplemental Table 3).

### Characteristics Before PSM

Before matching, there was a statistically significant difference in age among four groups: group [2,15] was the oldest and group (18,22] was the youngest. Compared with the other three groups, the incidence of MFS was lowest in the group [2,15] (*p* = 0.036). The incidence of this ABO was highest (80 (24.3%), *p* < 0.001) and traditional TAR+FET was lowest [241 (73.3%), *p* < 0.001] and statistically significant in group [2,15]. From group [2,15] to (22,73], the CPB time and HCA time were gradually prolonged, and an increase was therefore seen in surgical mortality rate (*p* = 0.014). There was statistically significant difference in ICU stay time among four groups, which was shortest in group [2,15] (median [IQR], 4.0 [3.0, 5.8] days) and longest in group (22,73] (5.0 [4.0, 6.4] days) with *p* < 0.001. There were also significant differences in the use of postoperative blood products; with the prolongation of HCA time, the use of blood products tended to increase (Supplemental Tables 1–3).

### Characteristics After PSM

**Figure 1:**
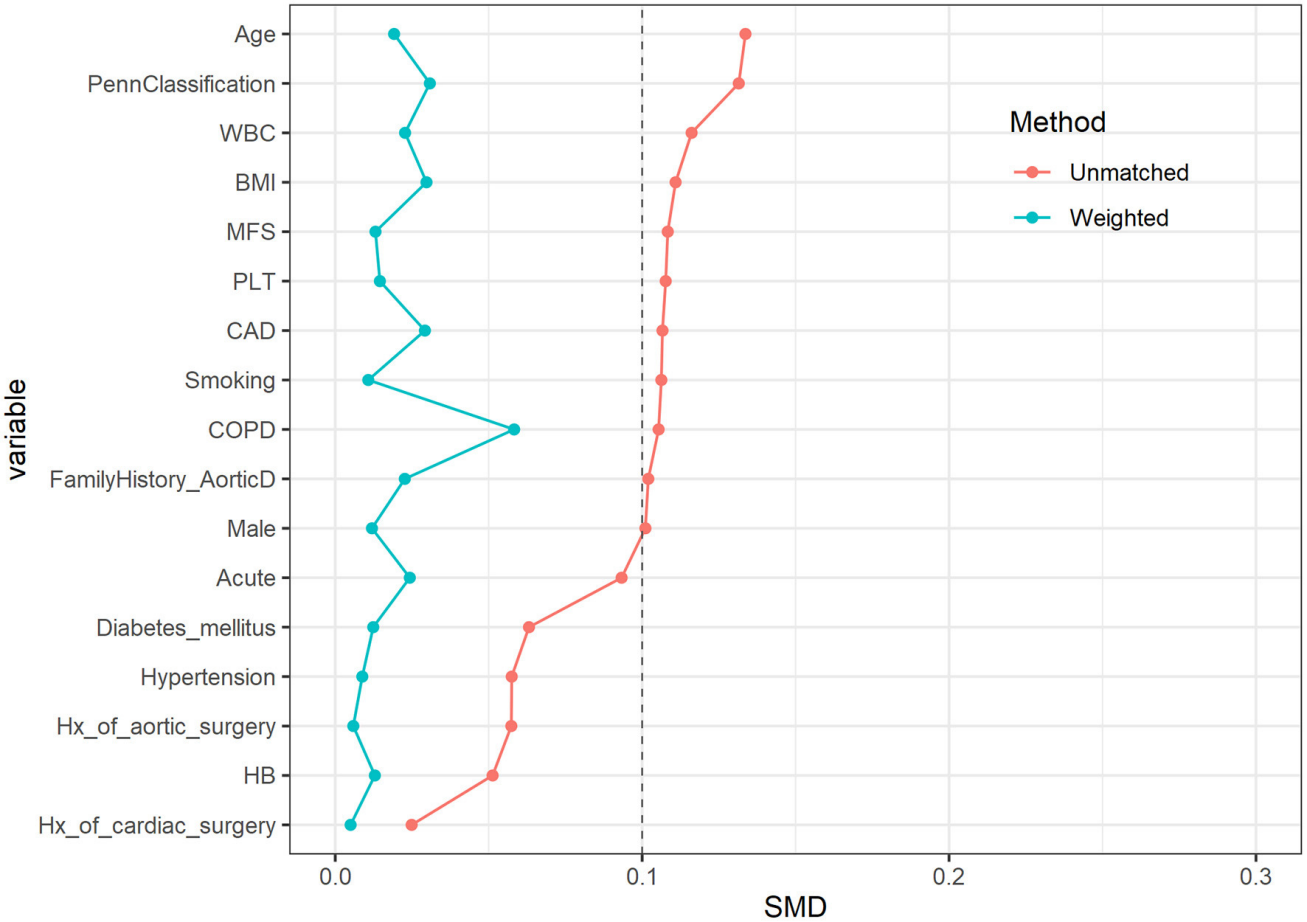
Love plot before and after propensity score matching (PSM) among characteristics. The distribution of main variables was stable after matching.

After PSM, the number of patients effectively matched in four groups was 1,015.9, 1,012.2, 1,015.0, and 1,010.1, respectively. There was no statistical difference between the four groups in preoperative characteristics; the p-value was close to 1. The distribution of main variables was stable after matching; the Love Plot is shown in Figure 1.

While focusing on the operation data, there were statistical differences in the use of TAR+FET surgery and ABO auxiliary measure among the four groups. Group [2,15] used the most ABO (227.2 (22.4%), p <0.001), while the group performed the least TAR+FET surgery (763.7 (75.2%), p <0.001). While focusing on clinical outcomes, the surgical mortality rates of four groups were 4.1, 6.6, 7.8, and 10.9%, with *p* = 0.041, respectively. Group [2,15] has the shortest ICU stay time, while group (22,73] has the longest, which was significantly different (*p* < 0.001). There were also significant differences in the use of postoperative blood products; from the group [2,15] to (22,73], the volume of transfusions increased gradually (*p* < 0.05) (Tables 1–3).

**Table 1:** Preoperative characteristics after propensity score matching.

	**Overall**	**[2,15]**	**(15,18]**	**(18,22]**	**(22,73]**	***p***
**n**	**4,053.3**	**1,015.9**	**1,012.2**	**1,015**	**1 <010.1**	
Age [mean (SD)]	49.0 (11.3)	49.1 (12.3)	49.0 (11.1)	48.8 (10.7)	49.1 (11.1)	0.983
Male (%)	3,041.6 (75.0)	760.3 (74.8)	757.1 (74.8)	769.1 (75.8)	755.1 (74.8)	0.992
BMI [mean (SD)]	26.0 (4.5)	26.0 (4.3)	26.1 (4.7)	25.8 (4.1)	26.0 (4.9)	0.915
Hypertension (%)	3,246.7 (80.1)	816.4 (80.4)	813.4 (80.4)	810.2 (79.8)	806.8 (79.9)	0.998
Diabetes mellitus (%)	116.8 (2.9)	28.9 (2.8)	31.7 (3.1)	27.6 (2.7)	28.5 (2.8)	0.993
CAD (%)	109.9 (2.7)	28.8 (2.8)	23.9 (2.4)	32.4 (3.2)	24.8 (2.5)	0.935
COPD (%)	15.4 (0.4)	4.2 (0.4)	5.9 (0.6)	5.2 (0.5)	0.0 (0.0)	0.65
MFS (%)	367.0 (9.1)	90.5 (8.9)	90.0 (8.9)	97.3 (9.6)	89.2 (8.8)	0.991
Smoking (%)	1,673.0 (41.3)	416.3 (41.0)	414.5 (40.9)	425.4 (41.9)	416.8 (41.3)	0.996
Family History AD (%)	68.6 (1.7)	14.1 (1.4)	17.2 (1.7)	17.5 (1.7)	19.8 (2.0)	0.961
Hx of cardiac surgery (%)	208.6 (5.1)	52.8 (5.2)	53.1 (5.2)	51.6 (5.1)	51.1 (5.1)	1
Hx of aortic surgery (%)	205.5 (5.1)	50.6 (5.0)	50.4 (5.0)	52.6 (5.2)	51.9 (5.1)	0.999
Acute (%)	3,420.8 (84.4)	850.1 (83.7)	861.7 (85.1)	861.3 (84.9)	847.6 (83.9)	0.965
HB [mean (SD)]	135.8 (17.4)	135.6 (17.4)	135.9 (17.4)	136.0 (18.1)	135.8 (17.0)	0.995
WBC [mean (SD)]	11.4 (4.4)	11.4 (4.2)	11.4 (4.9)	11.3 (4.3)	11.3 (4.2)	0.967
PLT [mean (SD)]	194.9 (75.6)	195.7 (69.6)	195.4 (73.4)	194.7 (85.8)	193.7 (73.0)	0.992
Penn Classification (%)						1
Aa	3,028.4 (74.7)	761.3 (74.9)	755.4 (74.6)	756.1 (74.5)	755.5 (74.8)	
Ab	902.8 (22.3)	223.6 (22.0)	223.8 (22.1)	227.6 (22.4)	227.8 (22.6)	
Ac	93.0 (2.3)	22.0 (2.2)	26.3 (2.6)	23.8 (2.3)	20.9 (2.1)	
Abc	29.1 (0.7)	9.0 (0.9)	6.7 (0.7)	7.6 (0.7)	5.8 (0.6)	

*SD, standard deviation; BMI, body mass index; COPD, chronic obstructive pulmonary disease; MFS, Marfan's syndrome; AD, aortic disease; Hx, history; HB, hemoglobin; WBC, white blood cell; PLT, platelet*.

**Table 2:** Operative data after propensity score matching.

	**Overall**	**[2,15]**	**(15,18]**	**(18,22]**	**(22,73]**	***p***
**n**	**4,053.3**	**1,015.9**	**1,012.2**	**1,015**	**1,010.1**	
Root Operation (%)						0.771
Bentall	997.9 (24.6)	240.3 (23.7)	221.1 (21.8)	295.1 (29.1)	241.4 (23.9)	
Root sparing	2,956.2 (72.9)	743.3 (73.2)	769.4 (76.0)	696.3 (68.6)	747.2 (74.0)	
David	34.7 (0.9)	14.4 (1.4)	7.1 (0.7)	4.4 (0.4)	8.8 (0.9)	
Wheats	64.5 (1.6)	17.9 (1.8)	14.7 (1.5)	19.2 (1.9)	12.8 (1.3)	
CABG (%)	436.2 (10.8)	92.0 (9.1)	113.0 (11.2)	104.0 (10.2)	127.2 (12.6)	0.664
TAR (%)	85.8 (2.1)	25.1 (2.5)	8.1 (0.8)	19.3 (1.9)	33.3 (3.3)	0.299
TAR_FET (%)	3,740.3 (92.3)	763.7 (75.2)	1,004.1 (99.2)	995.7 (98.1)	976.8 (96.7)	<0.001
ABO (%)	227.2 (5.6)	227.2 (22.4)	0.0 (0.0)	0.0 (0.0)	0.0 (0.0)	<0.001
Asc Iliac Bypass (%)	225.6 (5.6)	68.4 (6.7)	55.5 (5.5)	52.3 (5.2)	49.4 (4.9)	0.822
Operation time hour [mean (SD)]	6.2 (1.6)	6.1 (1.8)	6.1 (1.7)	6.2 (1.6)	6.5 (1.4)	0.005
CPB [mean (SD)]	172.5 (47.3)	160.3 (48.7)	165.7 (46.6)	176.7 (43.2)	187.3 (46.2)	<0.001
ACC [mean (SD)]	101.5 (33.1)	98.1 (37.0)	97.6 (34.6)	101.4 (26.8)	108.7 (32.2)	0.001
HCA [mean (SD)]	19.1 (6.8)	11.5 (3.9)	16.9 (0.8)	20.3 (1.1)	27.5 (5.5)	<0.001
Nadir Temp Nasa [mean (SD)]	22.5 (3.5)	24.4 (2.9)	23.1 (3.3)	22.2 (3.5)	20.2 (2.9)	<0.001

*SD, standard deviation; CABG, coronary artery bypass graft; TAR, total arch replacement; FET, frozen elephant trunk; ABO, aortic balloon occlusion; Asc Iliac Bypass, ascending-iliac bypass; CPB, cardiopulmonary bypass; ACC, aortic cross-clamp; HCA, hypothermic cardiac arrest; Nadir Temp Nasa, temperature of nadir nasopharyngeal*.

**Table 3:** Outcome data after matching.

	**Overall**	**[2,15]**	**(15,18]**	**(18,22]**	**(22,73]**	***p***
**n**	**4,053.3**	**1,015.9**	**1,012.2**	**1,015**	**1,010.1**	
**CMO (%)**	639.7(15.8)	126.7(12.5)	177.2(17.5)	142.0(14.0)	193.8(19.2)	0.171
Early Mortality (%)	298.5 (7.4)	41.5 (4.1)	67.2 (6.6)	79.4 (7.8)	110.4 (10.9)	0.041
Stroke (%)	107.3 (2.6)	35.7 (3.5)	22.2 (2.2)	13.1 (1.3)	36.3 (3.6)	0.301
Paralysis (%)	136.9 (3.4)	36.2 (3.6)	41.5 (4.1)	30.3 (3.0)	28.9 (2.9)	0.879
CRRT (%)	340.5 (8.4)	64.1 (6.3)	100.4 (9.9)	78.3 (7.7)	97.7 (9.7)	0.445
Heart failure (%)	114.4 (2.8)	32.4 (3.2)	38.4 (3.8)	37.0 (3.6)	6.6 (0.7)	0.117
Pneumonia (%)	832.4 (20.5)	236.6 (23.3)	174.3 (17.2)	188.9 (18.6)	232.6 (23.0)	0.253
Reoperation for bleeding (%)	149.5 (3.7)	44.9 (4.4)	36.5 (3.6)	46.1 (4.5)	22.0 (2.2)	0.508
Tracheotomy (%)	136.4 (3.4)	44.9 (4.4)	30.2 (3.0)	24.1 (2.4)	37.3 (3.7)	0.623
MODS (%)	50.8 (1.3)	8.6 (0.8)	8.4 (0.8)	14.7 (1.4)	19.1 (1.9)	0.678
Hospital Stay day (median [IQR])	12.0 [10.0, 17.0]	12.0 [9.0, 16.0]	12.0 [10.0, 18.0]	13.0 [10.0, 17.0]	13.0 [10.0, 17.0]	0.031
ICU Stay day (median [IQR])	4.7 [3.6, 6.3]	4.0 [2.8, 5.7]	4.7 [3.0, 6.7]	4.8 [3.6, 6.5]	5.0 [4.0, 6.4]	<0.001
Blood loss (ml) (median [IQR])	762.9 [600.0, 1,000.0]	690.0 [600.0, 900.0]	690.0 [600.0, 1,050.0]	780.0 [600.0, 1,050.0]	758.0 [600.0, 1,200.0]	0.306
RBC Transfusion (median [IQR])	4.0 [0.0, 7.9]	2.0 [0.0, 6.0]	4.0 [0.0, 6.0]	4.0 [0.0, 6.0]	4.0 [0.0, 8.0]	0.025
Plasma Transfusion (median [IQR])	400.0 [0.0, 800.0]	400.0 [0.0, 600.0]	400.0 [0.0, 800.0]	400.0 [0.0, 800.0]	600.0 [0.0, 800.0]	0.011
PLT Transfusion (median [IQR])	3.0 [1.0, 4.0]	1.0 [1.0, 3.0]	2.0 [1.0, 4.0]	3.3 [1.0, 4.0]	4.0 [3.0, 4.0]	<0.001

*IQR, interquartile range; CMO, composite major outcomes; CRRT, continuous renal replacement therapy; ICU, intensive care unit; RBC, red blood cell; PLT, platelet*.

### Restricted Cubic Spline Outcome

**Figure 2:**
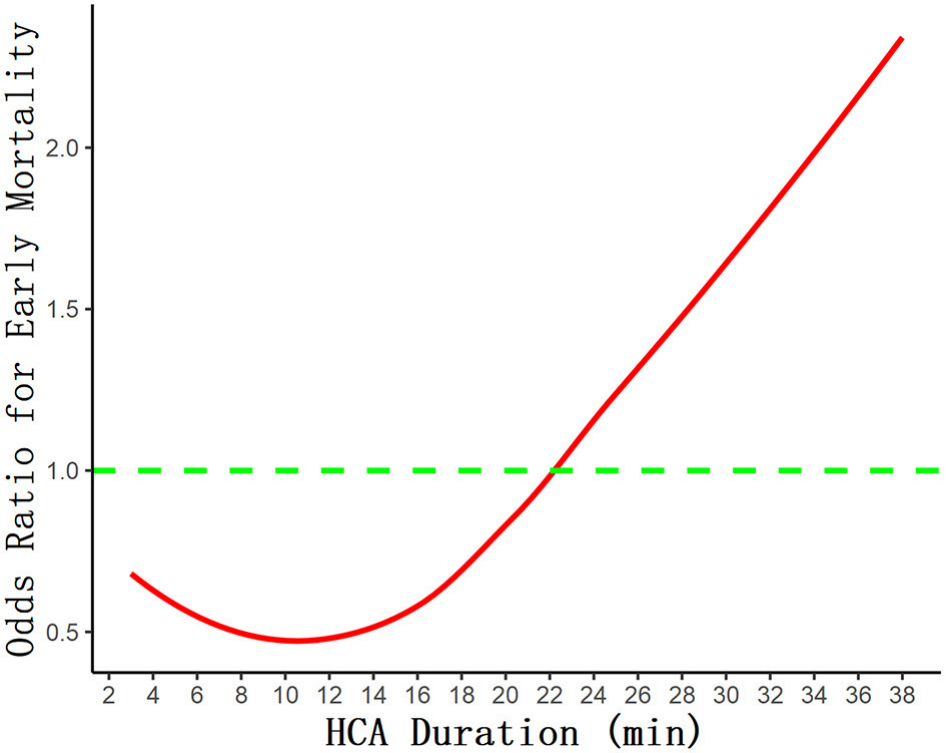
The association between the HCA time and surgical mortality was evaluated with restricted cubic spline curve based on Cox proportional hazards models.

In Figure 2, the association between the HCA time and surgical mortality was evaluated with an RCS curve based on Cox proportional hazards models. As shown in the curve, the risk of early mortality was relatively flat until HCA time reached 22 min approximately, then started to increase rapidly afterward. The risk is lowest when the HCA time is controlled at 8–14 min.

### Subgroup Analysis

**Figure 3:**
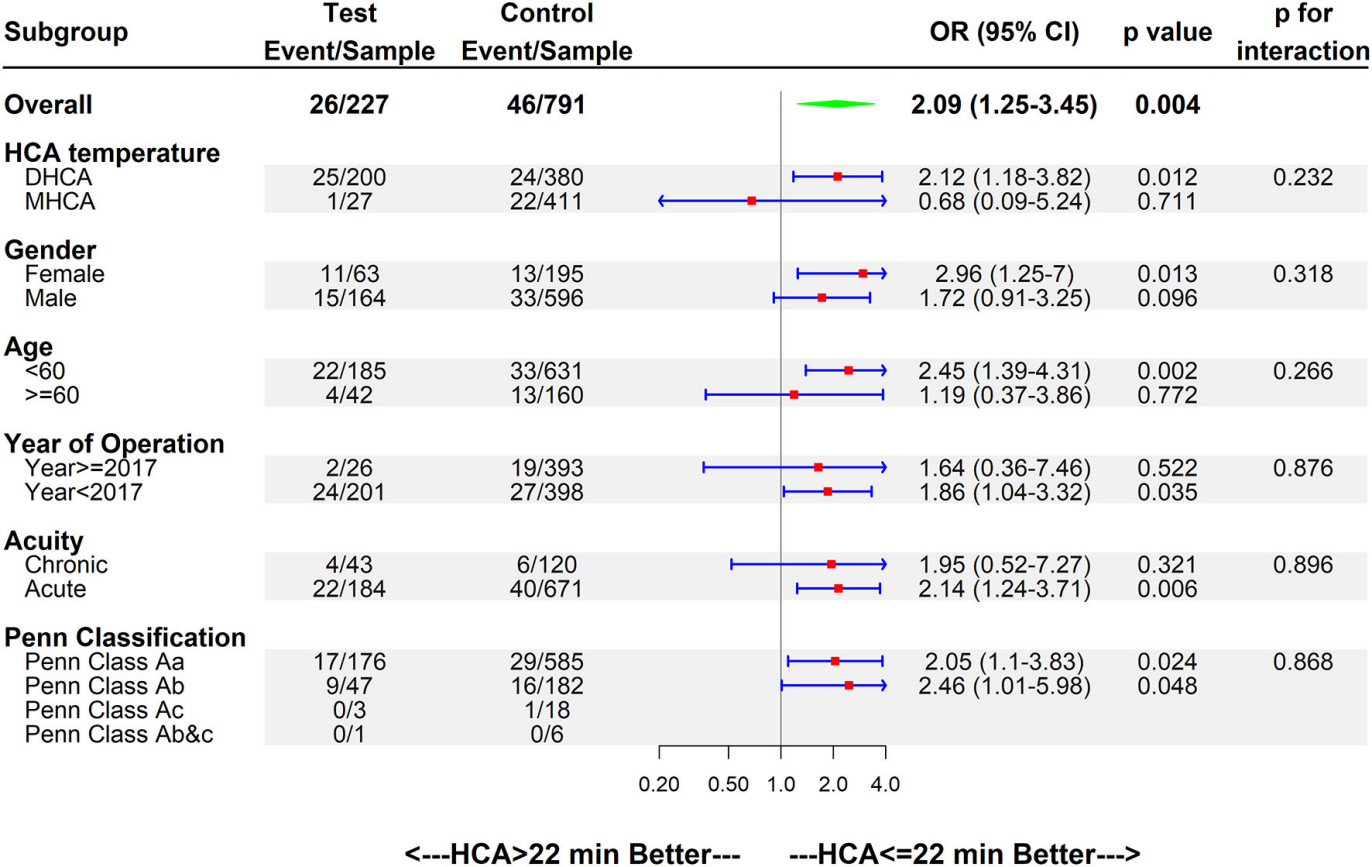
Forest plot of HCA>22 min better vs. HCA ≤ 22 min better regarding early mortality by subgroups. OR, odds ratio; CI, confidence interval.

Overall, the HCA time of 22 min or less was associated with better clinical outcomes (OR 2.09, 95%CI 1.25–3.45, p = 0.004); the data of subgroup analysis are shown in Figure 3. For the use of the DHCA strategy, HCA time ≤ 22 min was associated with better clinical outcomes (OR 2.12, 95%CI 1.18–3.82, *p* = 0.012). For women (OR 2.96, 95%CI 1.25–7.00, *p* = 0.012), age <60 (OR 2.45, 95%CI 1.39–4.31, *p* = 0.013), and in the acute phase (OR 2.14, 95%CI 1.24–3.71, *p* = 0.006), the HCA time of 22 min or less is associated with better clinical outcomes. Controlling HCA time ≤ 22 min can lead to better clinical outcomes in patients with preoperative malperfusion, regardless of the Penn classification (p <0.05).

## Discussion

The application of the HCA technique can provide a relatively bloodless field of operation for arch surgery; meanwhile, it aims to protect the brain and other terminal organs. In 1975, Dr. Griepp and his colleagues developed the HCA technique and performed aortic arch replacement surgery. Satisfying clinical results were obtained, and the safety and feasibility of this technique had been proved as well (18). In the following decades, HCA has become an important part of arch surgery, especially TAR or TAR+FET, and has been widely used and studied in animal experiments and human clinical work (19, 20). However, the optimal control of HCA time is still under discussion. In our center, through a statistical study of 1,018 patients with PSM and RCS, we concluded that early mortality increased with the prolongation of HCA time, as well as increased length of ICU stay and the use of blood products.

It is one-sided to talk about “duration” apart from “temperature” for HCA technology. A retrospective study focus on aortic arch surgery with HCA and ACP showed that a high temperature (24.1–28.0°C) is more effective in providing cerebral protection than a low temperature (≤24.0°C) in patients for <40 min, and suggested that the high-temperature group was an independent risk factor for 30-day mortality (21). In our center, the 20–28°C temperature strategy was mainly adopted; combined with the data in our study, the early mortality rate was 7.1%, which was significantly lower than those in other studies.

With the improvement of surgical techniques and equipment, the temperature of HCA has gradually increased (20–28°C), but the safe time range for HCA is still very different and controversial. Perreas et al. studied the use of DHCA+RCP (retrograde cerebral perfusion) in hemi-arch replacement, whose results showed that duration of DHCA is the only and main factor related to death (22). Their 30-day mortality rate was 9.6%, compared with 7.4% in our study, and they were also grouped according to different HCA times with mean HCA time 25.4 ± 13 min and RCP brain protection. As the grouping strategy of this study is based on a greatly different time span from ours, the specific mortality rates were not comparable. The rate of mortality and stroke in this study was lower than that of ours, of which a possible reason is that surgeries in our study were mainly TARs, which can be more complex and extensive, instead of hemi-arch replacements. As for stroke, although there was no statistically significant difference between the four groups, there was a trend of the incidence gradually increasing with the prolongation of HCA time, which is consistent with O'Hara's study (23). According to their report, in multivariable analysis, death or stroke risk increased with longer HCA time (OR 1.11 per 10-min increment, 95% CI 1.08–1.14). And at 30–40 min of HCA with ACP, the incidence of stroke was 11.4%, which was significantly higher than that in our cohort. It is generally recognized that there should be a negative correlation between the duration of HCA and the prognosis of patients. Early mortality, stroke, and renal function impairment increased significantly in patients with HCA time exceeding 40 min or even 50 min (24). Currently, conventional TAR or hemi-arch replacement procedures are generally able to control the HCA time within 30 min in patients with uncomplicated lesions, but it is important to know the optimal cutoff time and how the risk changes over time. As was shown on the RCS curve (Figure 2), the risk of early mortality was relatively flat until HCA time reached 22 min approximately, then started to increase rapidly afterward, which is consistent with the results of Immer et al. (10).

The application of HCA technology also has non-negligible effects on coagulation function. Studies have reported that when the core temperature is lower than 35°C, the number and function of platelets will be affected to a certain extent, and when the core temperature is lower than 33°C, other steps in the coagulation cascade reaction, such as the synthesis and kinetics of clotting enzymes and plasminogen activator inhibitors, may also be affected (19, 25). It follows from this that, in many studies, HCA has been associated with increased chest tube output and reintervention for bleeding (26). From our data, it can be seen that the rate of reoperation for bleeding and blood loss did not increase significantly with the prolongation of HCA time, which may be explained by the difference in surgical methods in different groups. In group (18,22], the reoperation for bleeding rate (4.5%) and blood loss (780.0 [600.0, 1,050.0]ml) were slightly higher than those in the other three groups, which is possible because there was a higher proportion of Bentall and Wheat's operations in this group that was difficult in hemostasis of the aortic root and sometimes did not wrap the prosthesis. Although there was no significant difference in blood loss, the volume of transfusions increased gradually (*p* < 0.05) with the prolongation of HCA time. In addition, our data showed that there was no significant correlation between stroke, paralysis, and the use of continuous renal replacement therapy (CRRT) and the duration of HCA (Table 3).

Currently, our center has been using HCA with a core temperature of 20–28°C for TAR surgery for many years, and the HCA time can be controlled within 20 min in most cases while satisfying clinical effects can be obtained. However, we expect that with the innovation of surgical techniques and instruments, the HCA time and temperature will be further improved. For example, our center has developed a “sutureless integrated stented graft” (SIS graft) device, with which a significantly shortened HCA time of 5–10 min, as well as a higher core temperature of 28–30°C, can be reached. This device is currently undergoing clinical trials and may further benefit patients with TAAD (27).

### Limitations

The study is observational and retrospective in nature. Our study is based on the overall situation of the medical facilities in our center, the surgeons, anesthesiologists, and ICU physicians, as well as the patients' condition in the region, so there may be differences between the results of our study and those of other centers. In addition, different centers have different definitions of HCA temperature (DHCA or MHCA). However, we performed a variety of statistical methods, such as PSM, RCS, and subgroup analysis, to reduce the impact of potential bias. We look forward to prospective controlled trials with larger sample sizes and more reasonable results.

### Conclusion

When arch surgery is performed, it is safe to keep the HCA time to <22 min from our single-center experience, at a core temperature of 20–28°C. Beyond this range, the early mortality increases significantly with the duration of HCA time. Of course, the degree of aortic dissection lesion and the complexity of surgical procedures must also be considered. The HCA time needs to be further controlled to bring a better early prognosis for patients.

## Data Availability Statement

The raw data supporting the conclusions of this article will be made available by the authors, without undue reservation.

## Ethics Statement

The studies involving human participants were reviewed and approved by Ethics Committee of Fuwai Hospital (ChiECRCT-20180041). Written informed consent for participation was not required for this study in accordance with the national legislation and the institutional requirements.

## Author Contributions

JS, JW, and JuQ developed study design. JS wrote the paper. JiQ, FC, WG, RZ, LD, SF, and EX organized patient recruitment and collected study statistics. JW involved in the statistical analyses and diagramming. XS, XQ, BW, WW, DW, XL, and CY supervised and provided guidance to the whole research process. All authors contributed to the article and approved the submitted version.

## Funding

This study was supported by Beijing Science and Technology Program (No. Z191100007619042), Capital Health Development and Scientific Research Foundation (No. 2018-2-4035), and the National Key Research and Development Program (No. 2018YFB1107102).

## Conflict of Interest

The authors declare that the research was conducted in the absence of any commercial or financial relationships that could be construed as a potential conflict of interest.

## Publisher's Note

All claims expressed in this article are solely those of the authors and do not necessarily represent those of their affiliated organizations, or those of the publisher, the editors and the reviewers. Any product that may be evaluated in this article, or claim that may be made by its manufacturer, is not guaranteed or endorsed by the publisher.

## Abbreviations

HCA: hypothermia circulatory arrest
TAAD: type A aortic dissection
TAR: total arch replacement
ABO: aortic balloon occlusion
DHCA: deep hypothermic cardiac arrest
MHCA: moderate hypothermic cardiac arrest
ACP: antegrade cerebral perfusion
RCP: retrograde cerebral perfusion
CRRT: continuous renal replacement therapy.
