# Supplementary material for: It Is Advisable to Control the Duration of Hypothermia Circulatory Arrest During Aortic Dissection Surgery: Single-Center Experience
Source: Front Cardiovasc Med. 2021 Dec 10;8:773268. doi: 10.3389/fcvm.2021.773268 (PMC8702722; doi:10.3389/fcvm.2021.773268)
Supplement: Supplementary file 1 [file Table_1.DOCX]

**Supplemental Table 1. Preoperative characteristics before propensity score matching.**

|  | Overall | [2,15] | (15,18] | (18,22] | (22,73] | p |
| --- | --- | --- | --- | --- | --- | --- |
| n | 1018 | 329 | 246 | 216 | 227 |  |
| Age (mean (SD)) | 49.1 (11.4) | 50.6 (12.0) | 48.3 (11.1) | 47.8 (10.9) | 49.1 (11.3) | 0.022 |
| Male (%) | 760 (74.7) | 239 (72.6) | 198 (80.5) | 159 (73.6) | 164 (72.2) | 0.114 |
| BMI (mean (SD)) | 26.0 (4.5) | 26.2 (4.2) | 26.3 (4.9) | 25.4 (4.2) | 25.8 (4.8) | 0.14 |
| Hypertension (%) | 814 (80.0) | 271 (82.4) | 192 (78.0) | 172 (79.6) | 179 (78.9) | 0.585 |
| Diabetes mellitus (%) | 30 (2.9) | 12 (3.6) | 8 (3.3) | 6 (2.8) | 4 (1.8) | 0.619 |
| CAD (%) | 28 (2.8) | 8 (2.4) | 3 (1.2) | 7 (3.2) | 10 (4.4) | 0.187 |
| COPD (%) | 6 (0.6) | 1 (0.3) | 4 (1.6) | 1 (0.5) | 0 (0.0) | 0.095 |
| MFS (%) | 91 (8.9) | 17 (5.2) | 26 (10.6) | 23 (10.6) | 25 (11.0) | 0.036 |
| Smoking (%) | 421 (41.4) | 144 (43.8) | 109 (44.3) | 89 (41.2) | 79 (34.8) | 0.127 |
| Family History AD (%) | 20 (2.0) | 2 (0.6) | 5 (2.0) | 6 (2.8) | 7 (3.1) | 0.147 |
| Hx of cardiac surgery (%) | 53 (5.2) | 17 (5.2) | 14 (5.7) | 10 (4.6) | 12 (5.3) | 0.966 |
| Hx of aortic surgery (%) | 53 (5.2) | 17 (5.2) | 10 (4.1) | 11 (5.1) | 15 (6.6) | 0.669 |
| Acute (%) | 855 (84.0) | 285 (86.6) | 210 (85.4) | 176 (81.5) | 184 (81.1) | 0.211 |
| HB (mean (SD)) | 135.7 (17.4) | 136.4 (16.9) | 135.8 (17.5) | 135.4 (18.1) | 134.7 (17.5) | 0.735 |
| WBC (mean (SD)) | 11.4 (4.9) | 11.6 (4.1) | 11.9 (6.6) | 11.0 (4.3) | 11.0 (4.1) | 0.09 |
| PLT (mean (SD)) | 195.5 (78.4) | 192.4 (67.8) | 193.0 (71.4) | 208.6 (104.0) | 190.3 (70.7) | 0.049 |
| Penn Classification (%) |  |  |  |  |  | 0.589 |
| Aa | 761 (74.8) | 247 (75.1) | 184 (74.8) | 154 (71.3) | 176 (77.5) |  |
| Ab | 229 (22.5) | 76 (23.1) | 51 (20.7) | 55 (25.5) | 47 (20.7) |  |
| Ac | 21 (2.1) | 4 (1.2) | 9 (3.7) | 5 (2.3) | 3 (1.3) |  |
| Abc | 7 (0.7) | 2 (0.6) | 2 (0.8) | 2 (0.9) | 1 (0.4) |  |

SD, standard deviation; BMI, body mass index; CAD, coronary artery disease; COPD, chronic obstructive pulmonary disease; MFS, Marfan’s syndrome; AD, aortic disease; Hx, history; HB, hemoglobin; WBC, white blood cell; PLT, platelet.
