# Supplementary material for: It Is Advisable to Control the Duration of Hypothermia Circulatory Arrest During Aortic Dissection Surgery: Single-Center Experience
Source: Front Cardiovasc Med. 2021 Dec 10;8:773268. doi: 10.3389/fcvm.2021.773268 (PMC8702722; doi:10.3389/fcvm.2021.773268)
Supplement: Supplementary file 2 [file Table_2.DOCX]

**Supplemental Table 2. Operative data before propensity score matching.**

|  | Overall | [2,15] | (15,18] | (18,22] | (22,73] | p |
| --- | --- | --- | --- | --- | --- | --- |
| n | 1018 | 329 | 246 | 216 | 227 |  |
| Root Operation (%) |  |  |  |  |  | 0.571 |
| Bentall | 253 (24.9) | 73 (22.2) | 56 (22.8) | 66 (30.6) | 58 (25.6) |  |
| Root sparing | 739 (72.6) | 246 (74.8) | 185 (75.2) | 144 (66.7) | 164 (72.2) |  |
| David | 9 (0.9) | 4 (1.2) | 2 (0.8) | 1 (0.5) | 2 (0.9) |  |
| Wheats | 17 (1.7) | 6 (1.8) | 3 (1.2) | 5 (2.3) | 3 (1.3) |  |
| CABG (%) | 106 (10.4) | 30 (9.1) | 27 (11.0) | 19 (8.8) | 30 (13.2) | 0.363 |
| TAR (%) | 22 (2.2) | 8 (2.4) | 2 (0.8) | 4 (1.9) | 8 (3.5) | 0.229 |
| TAR_FET (%) | 916 (90.0) | 241 (73.3) | 244 (99.2) | 212 (98.1) | 219 (96.5) | <0.001 |
| ABO (%) | 80 (7.9) | 80 (24.3) | 0 (0.0) | 0 (0.0) | 0 (0.0) | <0.001 |
| Asc Iliac Bypass (%) | 59 (5.8) | 22 (6.7) | 13 (5.3) | 12 (5.6) | 12 (5.3) | 0.867 |
| Operation time hour (mean (SD)) | 6.2 (1.7) | 6.1 (1.8) | 6.1 (1.8) | 6.1 (1.6) | 6.6 (1.5) | 0.009 |
| CPB (mean (SD)) | 171.8 (47.6) | 161.9 (48.6) | 166.6 (46.4) | 175.0 (43.5) | 188.9 (46.5) | <0.001 |
| ACC (mean (SD)) | 101.8 (33.8) | 99.6 (36.9) | 98.2 (35.0) | 100.6 (26.0) | 109.9 (33.2) | 0.001 |
| HCA (mean (SD)) | 18.3 (7.2) | 11.4 (4.0) | 16.9 (0.8) | 20.3 (1.1) | 27.9 (6.3) | <0.001 |
| Nadir Temp Nasa (mean (SD)) | 22.8 (3.5) | 24.6 (2.9) | 23.1 (3.4) | 22.2 (3.5) | 20.2 (2.9) | <0.001 |

SD, standard deviation; CABG, coronary artery bypass graft; TAR, total arch replacement; FET, frozen elephant trunk; ABO, aortic balloon occlusion; Asc Iliac Bypass, ascending-iliac bypass; CPB, cardiopulmonary bypass; ACC, aortic cross clamp; HCA, hypothermic cardiac arrest; Nadir Temp Nasa: temperature of nadir nasopharyngeal.
