# Supplementary material for: It Is Advisable to Control the Duration of Hypothermia Circulatory Arrest During Aortic Dissection Surgery: Single-Center Experience
Source: Front Cardiovasc Med. 2021 Dec 10;8:773268. doi: 10.3389/fcvm.2021.773268 (PMC8702722; doi:10.3389/fcvm.2021.773268)
Supplement: Supplementary file 3 [file Table_3.DOCX]

**Supplemental Table 3. Outcome data before propensity score matching.**

|  | Overall | [2,15] | (15,18] | (18,22] | (22,73] | p |
| --- | --- | --- | --- | --- | --- | --- |
| n | 1018 | 329 | 246 | 216 | 227 |  |
| **CMO (%)** | 160(15.7) | 43(13.1) | 42(17.1) | 28(13.0) | 47(20.7) | 0.055 |
| Early Mortality (%) | 72 (7.1) | 14 (4.3) | 17 (6.9) | 15 (6.9) | 26 (11.5) | 0.014 |
| Stroke (%) | 30 (2.9) | 13 (4.0) | 6 (2.4) | 3 (1.4) | 8 (3.5) | 0.323 |
| Paralysis (%) | 33 (3.2) | 11 (3.3) | 9 (3.7) | 6 (2.8) | 7 (3.1) | 0.957 |
| CRRT (%) | 85 (8.3) | 23 (7.0) | 22 (8.9) | 15 (6.9) | 25 (11.0) | 0.312 |
| Heart failure (%) | 30 (2.9) | 11 (3.3) | 10 (4.1) | 7 (3.2) | 2 (0.9) | 0.194 |
| Pneumonia (%) | 217 (21.3) | 82 (24.9) | 43 (17.5) | 40 (18.5) | 52 (22.9) | 0.109 |
| Reoperation for bleeding (%) | 38 (3.7) | 15 (4.6) | 9 (3.7) | 9 (4.2) | 5 (2.2) | 0.528 |
| Tracheotomy (%) | 37 (3.6) | 16 (4.9) | 7 (2.8) | 6 (2.8) | 8 (3.5) | 0.509 |
| MODS (%) | 13 (1.3) | 3 (0.9) | 2 (0.8) | 3 (1.4) | 5 (2.2) | 0.506 |
| Hospital Stay day (median [IQR]) | 12.0 [10.0, 17.0] | 11.0 [9.0, 16.0] | 12.0 [10.0, 18.0] | 13.0 [10.0, 17.0] | 13.0 [10.0, 17.5] | 0.003 |
| ICU Stay day (median [IQR]) | 4.7 [3.5, 6.0] | 4.0 [3.0, 5.8] | 4.7 [3.5, 6.7] | 4.8 [3.7, 6.0] | 5.0 [4.0, 6.4] | <0.001 |
| Blood loss (ml) (median [IQR]) | 750.0 [600.0, 960.0] | 690.0 [600.0, 900.0] | 690.0 [600.0, 1027.5] | 780.0 [600.0, 1050.0] | 780.0 [600.0, 1200.0] | 0.424 |
| RBC Transfusion (median [IQR]) | 4.0 [0.0, 6.0] | 2.0 [0.0, 6.0] | 4.0 [0.0, 6.0] | 4.0 [0.0, 6.0] | 4.0 [0.0, 8.0] | 0.005 |
| Plasma Transfusion (median [IQR]) | 400.0 [0.0, 800.0] | 400.0 [0.0, 688.0] | 500.0 [0.0, 800.0] | 400.0 [0.0, 800.0] | 600.0 [0.0, 800.0] | 0.026 |
| PLT Transfusion (median [IQR]) | 3.0 [1.0, 4.0] | 1.0 [1.0, 3.0] | 2.0 [1.0, 4.0] | 3.0 [1.0, 4.0] | 4.0 [3.0, 4.0] | <0.001 |

IQR, interquartile range; CMO, composite major outcomes; CRRT, continuous renal replacement therapy; ICU, intensive care unit; RBC, red blood cell; PLT, platelet.
